# Identification of robust species-specific marker genes for the demarcation of the Klebsiella pneumoniae–quasipneumoniae–variicola complex

**DOI:** 10.1099/jmm.0.002102

**Published:** 2025-11-17

**Authors:** Mustafa Galib, Viet Hung Nguyen

**Affiliations:** 1 Department of Mathematics and Natural Sciences, BRAC University, Dhaka, Bangladesh; 2 Project Genomes To Functional, Ecological, and Evolutionary Characterizations (Project G2FEEC), Ho Chi Minh City, Vietnam

**Keywords:** bacterial phylogeny, bacterial taxonomy, KEGG Orthology, *Klebsiella* species, species-specific biomarkers, whole-genome sequencing

## Abstract

**Introduction.** *Klebsiella pneumoniae*, *Klebsiella quasipneumoniae* and *Klebsiella variicola* are closely related species (collectively referred to as ‘PQV’) but exhibit distinct clinical presentations and epidemiological profiles. These distinctions are important for proper diagnosis and treatment of infections and other *Klebsiella*-related diseases. The ability to differentiate between these species is becoming more important as we better understand their unique roles in disease.

**Hypothesis/Gap statement.** Despite the importance of accurate taxonomic classifications, both traditional and modern laboratory techniques fail to accurately delineate between the PQV species, which can lead to treatment failures. Conversely, taxonomy via whole-genome sequencing is highly accurate but is both costly and resource-intensive. Thus, there is a lack of a widely adopted method that balances both cost-effectiveness and accuracy for differentiating PQV species.

**Aim.** To assess the taxonomic accuracy of existing genome databases and classification tools for PQV species and to develop a rapid, cost-effective method for accurate species differentiation.

**Methodology.** To address these challenges, we extracted 78 representative PQV genomes from the Integrated Microbial Genomes and Microbiomes database. We used multiple comparative genomic comparison techniques, phylogenetic tree constructions and pangenome profiling to accurately phylogenetically and subsequently taxonomically classify the genomes. After establishing accurate taxonomic classifications, we identified species-specific marker genes (SSMGs) represented by KEGG Orthologies (KOs).

**Results.** The Genome Taxonomy Database (GTDB) classifications were consistent with our comparative genomic benchmarks, while the National Center for Biotechnology Informationdatabase originally contained six misclassified genomes. The GTDB-Tk tool also showed reliability for systematic classification of the genomes. Our KO-based screening identified 22 candidate SSMGs. Four markers (K05306, K07507, K13795 and K09955) exhibited significant specificity. ATP-binding proteins showed slightly higher maximum percentage identity values due to conserved domains but were still valuable within multi-locus SSMG panels.

**Conclusion.** Our findings establish the GTDB as the gold standard taxonomic reference for PQV classification when complete genomes are available. Additionally, we developed a practical panel of genetic markers that enables rapid, cost-effective and accurate species differentiation. These SSMGs represent practical tools that can be implemented in diagnostic laboratories for both clinical specimens and environmental surveillance, addressing a critical gap in clinical microbiology.

## Introduction

*Klebsiella* is a genus of bacteria that has received significant scientific and clinical attention due to its role in various infections [1]. Members of this genus can be found as part of the normal microbiota of the nose, throat, skin and intestinal tract of healthy individuals. However, they can also cause a wide range of opportunistic hospital- and community-acquired infections such as pneumonia, wound infections, urinary tract infections, bloodstream infections and sepsis [1].

*Klebsiella pneumoniae* in particular is part of the clinically significant ESKAPE pathogen group, which includes *Enterococcus faecium*, *Staphylococcus aureus*, *K. pneumoniae*, *Acinetobacter baumannii*, *Pseudomonas aeruginosa* and *Enterobacter* species [2,3]. These pathogens are hospital-associated pathogens that exhibit multi-resistance to antibiotics, thus pose a significant threat due to their ability to evade current treatments [2]. They exemplify antimicrobial resistance in both community and healthcare settings through convergent mechanisms, such as drug inactivation, target modification, reduced antibiotic accumulation, robust biofilm formation and horizontal gene transfer (HGT) [4]. *K. pneumoniae* is particularly associated with high levels of resistance to broad-spectrum antibiotics, including beta-lactams and carbapenems [5,8]. These organisms were designated as part of the ‘priority pathogens’ by the World Health Organization in 2017 due to the urgent need for new antimicrobial development and strategies to combat antibiotic resistance [4,5].

*K. pneumoniae* is a member of the *Klebsiella pneumoniae* species complex (KpSC), which has now been reclassified to comprise seven closely related species [9,10]. Among these, *K. pneumoniae, Klebsiella quasipneumoniae* and *Klebsiella variicola* are most prevalent globally, with notable reports from developing countries including Bangladesh and Vietnam [11,12]. They are also most phylogenetically closely related within the KpSC [7,13,15], collectively referred to as the ‘PQV’ in this study. Unsurprisingly, they do share genetic similarities that can lead to their misidentification in clinical settings, exacerbated by specific cases of intraspecific variations [14,16, 17]. This taxonomic ambiguity creates diagnostic challenges and complicates species identification, not only with traditional biochemical tests but also with more recent methods like matrix-assisted laser desorption/ionization time-of-flight, where inaccuracies often arise due to a lack of accurately curated reference databases [10,16, 18,23]. Amplicon sequencing has become more prevalent as an alternative; however, allelic mutations, chromosomal re-organizations, HGT and sequencing inaccuracies/biases can significantly impact accuracy even with multi-locus sequence typing assays [24,26].

The PQV species are known to differ in disease severity, antibiotic resistance and clinical outcomes, and thus, their misdiagnosis and subsequent mistreatment can be lethal [18,27]. For instance, a study in Sweden found that individuals infected with *K. variicola* had a significantly higher mortality rate than those with *K. pneumoniae* [28]. These differences in clinical impact are further complicated by extensive drug resistance (XDR), which is frequently observed in these bacteria. XDR features have become prevalent due to HGT [29], and rapid changes in both virulence factors and resistance mechanisms occur through chromosomal recombination and plasmid exchange [30]. These three species are also known for hypervirulent strains that have arisen independently multiple times and, therefore, also require tailored epidemiological surveillance and control strategies [31,32].

Currently, the most reliable method for identifying the various members of the PQV complex is whole-genome sequencing (WGS) [7,17]. However, WGS remains time-consuming and too costly to be widely accessible, especially in lower-income countries [33]. In addition, genome-based approaches can still face challenges due to inconsistencies in taxonomic protocols across different databases, such as between the Genome Taxonomy Database (GTDB) [34] and the National Center for Biotechnology Information (NCBI) database [35]. Even when manual curation aligns the NCBI with the GTDB, inconsistencies still continue to occur with new submissions to the NCBI. Additionally, updates are delayed while being propagated to other databases, such as the Integrated Microbial Genomes and Microbiomes (IMG/M) [36]. The challenge, therefore, is not only the robust species-level classification of PQV but also the need to do so at a reasonable time and financial cost.

This study has two main objectives. The first objective is a comprehensive phylogenetic analysis of PQV genomes using multiple genomic approaches to evaluate the accuracy of existing genome databases and genome-based classification tools to propose a gold standard going forward. The second objective is to develop an alternative method for rapidly and robustly delineating PQV species.

Although existing methodologies can reliably classify *Klebsiella* to the PQV complex, they face challenges in accurately distinguishing between the three individual species. Therefore, the challenge was developing a definitive approach to species-level identification that could serve as a new gold standard for rapid and robust differentiation of the PQV species. In this study, we identified species-specific marker genes (SSMGs) that distinguish between the three PQV species using a straightforward presence/absence criterion. Positive markers are genes present in all genomes of one species but absent in the other two species and vice versa for negative markers. This approach is conceptually similar to how CheckM uses biomarkers to determine genome completeness, but here, it is applied to delineate species rather than assess genome quality [37]. Sequencing of SSMGs can still be done via amplicon sequencing but is generally less affected by short sequence mutations or typical sequencing errors. However, careful primer design remains important.

Consequently, using SSMGs for identification can be significantly faster and more cost-efficient without sacrificing accuracy [38]. This approach is particularly valuable in both clinical diagnostics and environmental surveillance. It improves the efficiency and accuracy of PQV species differentiation. Such rapid species identification can inform timely intervention strategies while operating within resource constraints.

## Methods

### Data acquisition and genome selection

**Fig. 1.**
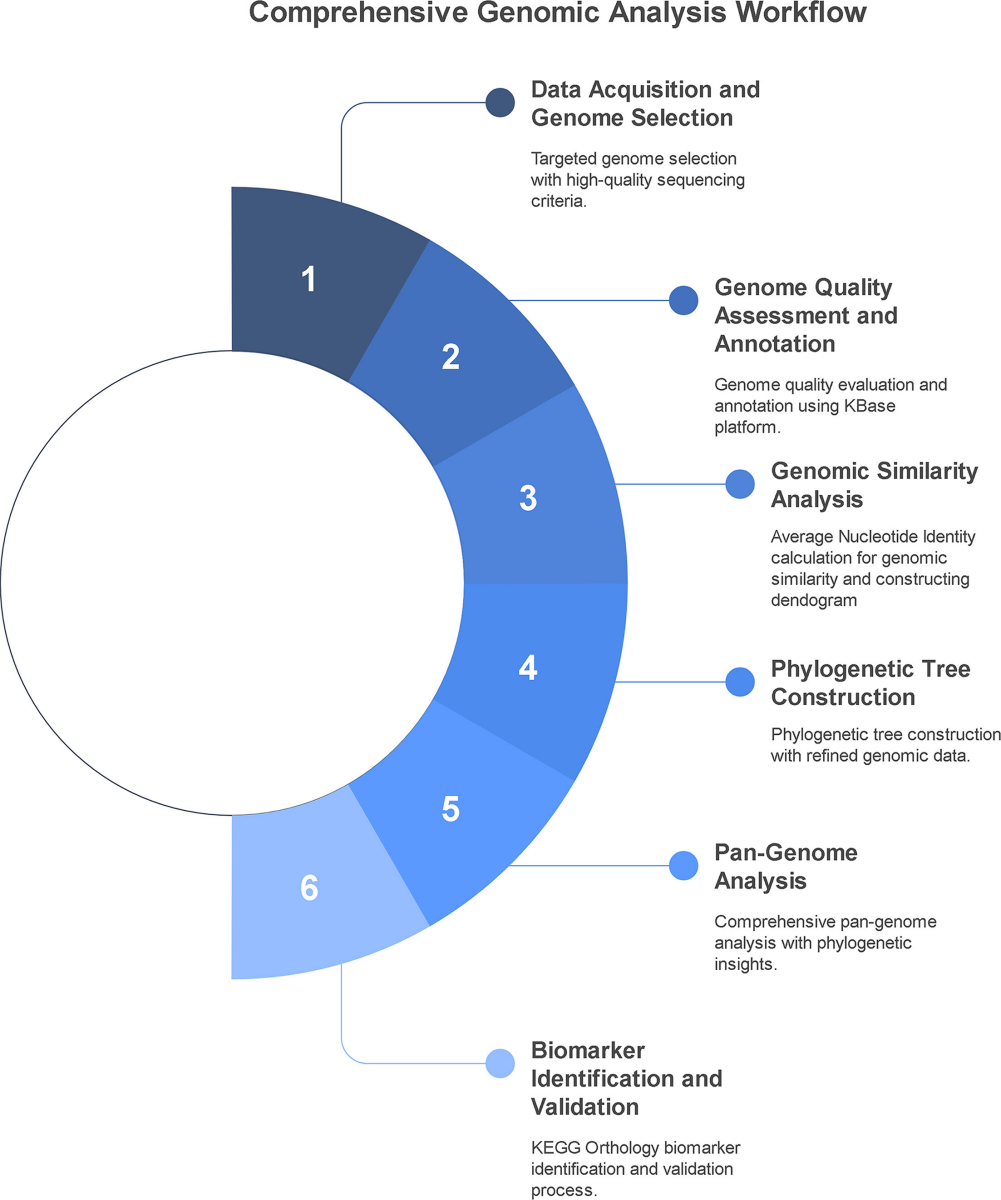
Workflow of comparative genomics analysis used in this study. The process includes six main steps: (1) genome data collection, (2) quality assessment and annotation, (3) genomic similarity analysis, (4) phylogenetic tree construction, (5) pangenome analysis and (6) identification and validation of species-specific markers.

We systematically identified PQV genomes using the IMG/M web service [36] with the ‘Advanced Search Builder’ tool. For each species, two separate searches were conducted: one with ‘Taxonomy’ set to ‘NCBI Species’ (according to the NCBI classification system) and the other to ‘GTDB Species’ (according to the Genome Taxonomy Database classification). High-quality, closed genomes were specifically required to identify delineating biomarkers, as we needed to ensure the absence of genomic elements was not due to assembly gaps or errors. Therefore, in all searches, the selection criterion of ‘Sequencing Quality’ was set to ‘Level 6’. The selected genomes were then cross-referenced to remove duplicates and ensure data consistency before being organized into a workspace within IMG/M. Genome quality (completeness and contamination) was evaluated using CheckM (version 1.0.18) [39]. The whole methodology is summarized in Fig. 1.

Three *Klebsiella oxytoca* genomes were chosen as the outgroup for all phylogenetic analyses. These *K. oxytoca* genomes were chosen because they are phylogenetically distinct from the *KpSC* but still within the *Klebsiella* genus [40]. This allowed them to act as stable anchors that improved phylogenetic tree rooting and enhanced the interpretation of evolutionary relationships.

### Species labelling

Genome accession IDs were used to retrieve corresponding taxonomic information and strain identifiers for consistent labelling throughout the manuscript. For each genome, the GTDB species name and the NCBI strain identifier were then extracted. With the taxonomic and strain identifiers extracted, a unique label was constructed for each genome using the format [*accession_id*]_[*GTDB_species_name*]_[*NCBI_strain_identifier*]. For example, the accession ID GCA_001598695.1, corresponding to *K. oxytoca* strain NBRC 105695, was labelled as ‘GCA_001598695.1_Klebsiella_oxytoca_NBRC_105695’. This systematic labelling approach helped to ensure clarity and traceability of all genomic data referenced, along with immediate comparisons to be drawn between the NCBI and GTDB databases.

### Species delineation

#### Whole-genome (multi-gene) and 16S rRNA phylogenetic analyses

##### Multi-gene phylogenetic analysis

The phylogenetic tree was constructed using the following workflow in the Department of Energy Systems Biology Knowledgebase (KBase) (v5.5.0) [41]:

Annotation: All genomes were first annotated with the Rapid Annotation using Subsystem Technology toolkit via the app ‘Annotate Multiple Microbial Assemblies with RASTtk’ (v1.073) [42,43].

Genome set preparation: A genome set was then prepared using the ‘Add Genomes to GenomeSet’ app (v1.7.6) [44].

Tree construction: The phylogenetic tree was constructed using the ‘Insert Set of Genomes into SpeciesTree’ app (v1.2.0) [45], which identified protein sequences from 49 conserved Clusters of Orthologous Genes (COGs) [46] and aligned them. The app then used the alignment file to construct the phylogenetic tree using FastTree2 [47].

Tree refinement: The tree was refined with the ‘Trim SpeciesTree to GenomeSet’ app (v1.4.0) [48] to remove duplicates.

Visualization: The tree was visualized with the Interactive Tree of Life (iTOL) web service[49] for rooting and re-formatting, including labelling.

Whole-genome phylogenetic classification (though more accurately, multi-gene phylogenetic classification) has become increasingly preferred over 16S rRNA phylogenetic classifications, especially in large-scale studies [50]. Even genes traditionally considered to have a relatively stable rate of mutation, like rRNA genes, can still have significant exceptions that produce important misclassifications, such as the case of *Shigella* and *Escherichia*, which were originally considered separate genera based on the distinctiveness of 16S rRNA genes, but the former has now been merged into the latter based on robust multi-gene phylogenetics [51]. In contrast, differing rates of mutations can be ‘smoothed’ across multiple genes, allowing for the impact of outlier rates of mutations to not skew phylogenetic determinations [51]. Phylogenetics of multiple conserved genes has been the basis of many robust taxonomic classifications, including that of the GTDB [34]. The specific methodology described above has previously been used for robust phylogenetic classification of diverse *Gammaproteobacteria* [52,54], including *Klebsiella* [55], and was, therefore, also applied here.

Separately, the Classify Microbes with the Genome Taxonomy Database Toolkit (GTDB-Tk) (v2.3.2) [56] application within KBase was also used to taxonomically classify all genomes. While this tool cannot infer phylogenetic relationships, it does produce robust taxonomic classifications, including those of *Klebsiella* [55] and was, therefore, valuable in robust validation of the other phylogenetic results in our study.

### 16S rRNA phylogenetic tree construction

The 16S rRNA gene tree was generated through the following process:

Sequence extraction: All 16S rRNA gene sequences were extracted for the 81 genomes from the IMG/M database [36] on 27 January 2025 and downloaded as a single fasta file containing 627 sequences.

Sequence alignment: The sequences were aligned using the muscle [57] (codons) algorithm in mega11 (v11.0.13) with default parameters [58].

Tree construction: A maximum likelihood tree was built in mega11 using the Jukes–Cantor model, 100 bootstrap replications and a uniform rate model. We set partial deletion to 95% to exclude positions with missing data in more than 5% of the sequences.

Rooting and formatting: The final tree was rooted and formatted using the mega11 phylogeny tool.

### Average nucleotide and amino acid identity clustering

Average nucleotide identity (ANI) is a particularly valuable metric in microbial taxonomy. It provides a quantitative measurement of genome-wide similarity that enables more precise species delineation, especially among closely related organisms [59,60], which is especially useful for species like PQV species [61,62]. The orthologous average nucleotide identity (OrthoANI) algorithm, in particular, specifically identifies and compares orthologous genes that are evolutionarily conserved and functionally equivalent across species [63,64]. This approach yields more robust pairwise identity calculations, and thus, we implemented it using the ‘pyorthoani’ tool. This tool efficiently calculates pairwise nucleotide identity between genomes using orthologous gene sets [65]. To further validate our findings from the ANI analysis, we also evaluated average amino acid identity (AAI) as an additional overall genome-relatedness index [66]. The EzAAI tool was used to both extract protein sequences from and then calculate the pair-wise AAI between each genome [67]. An in-house Python script was used to visualize the results of both the OrthoANI and AAI clusterings and to visualize them as dendrograms.

### Pangenome and phylogenetic analysis

#### Genome annotation

For the pangenome analysis, we first re-annotated the 81 genomes using Prokka (v1.14.6) [68] to generate standardized GFF3 files for each isolate. This was to ensure all genomes were analysed with the same software and parameters.

#### Pangenome construction with Roary

The re-annotated genomes were used to construct a pangenome with Roary (v3.13.0) [69] to identify core, accessory and unique genes across a set of genomes. This tool has been previously applied to differentiate pangenomic signatures between *K. pneumoniae* and *K. quasipneumoniae* [70]. In this study, we extended its use to differentiate between all three PQV species.

The specific parameters used for Roary were six parallel threads (-p 6), inclusion of all gene alignments (-e) and a minimum percentage identity via Basic Local Alignment Search Tool for proteins (blastp) of 95% (-i 95). A core gene definition requiring presence in ≥99% of genomes (-cd 99) and an MCL inflation value of 1.5 (-iv 1.5) to control gene cluster granularity was also used. Verbose output was enabled (-v), and the --mafft option was used to utilize MAFFT [71] for the core gene alignment. The output files were saved in the directory specified [69]. In addition to the combined analysis, we also re-ran the analysis excluding *K. oxytoca*, and then for each PQV species individually to provide a detailed view of species-specific pangenome compositions.

#### Phylogenetic tree construction

A phylogenetic tree was constructed from the core gene alignment file using FastTree (v2.1.11) [72]. The -nt parameter specified that the input data were nucleotide, while the -gtr and -gamma parameters were used to employ a generalized time-reversible model with a gamma distribution for rate variation, which are common and robust models for molecular evolution. We performed 100 bootstrap replicates (-boot 100) to assess branch support and used a thorough nearest-neighbour interchange search with eight rounds (-spr 8) to improve tree topology. The resulting phylogenetic tree was saved in a newick file format.

#### Pangenome analysis and visualization

We then used custom Python scripts to visualize the final pangenome data by generating a pangenome matrix and pangenome pie charts to effectively illustrate the distribution of core, accessory and unique genes.

#### KEGG Orthology biomarker (species-specific marker gene) identification

After the phylogenetic analyses confirmed the GTDB as a reliable taxonomic source and affirmed the GTDB-Tk [56] as a reliable taxonomic classification tool (detailed in the ‘Results and discussion’ section), the genomes were re-grouped based on this confirmed taxonomy. Due to the significantly higher number of *K. pneumoniae* genomes available, comparability can be skewed and yield results not equally reflective across all three PQV species analysed. Therefore, to ensure balanced comparability, we selected equal numbers of genomes for each PQV species, using all available high-quality genomes up to the limit set by the species with the fewest genomes.

For each genome, in the IMG/M, we accessed the ‘Statistics’ tab through the ‘Sample Name’ and extracted ‘Protein coding genes connected to KEGG Orthology (KO)’.' This data for all genomes was compiled into a comprehensive table. Using custom scripts, we identified KOs that were either present in all genomes of one species except one (‘100% in all but one’) or absent in all genomes of one species except one (‘0% in all but one’), which can then represent SSMGs.

There are a number of ways to classify annotated genes. Higher-level classifications are of protein families and domains, such as Pfams and TIGRFAMs [73,74], which represent categorical functional representations that do not necessarily demarcate different genes. Similarly, COGs [75] are groupings of similar genes. However, orthologs are not always functionally equivalent, especially for only distantly related orthologs. Enzyme commission (E.C.) numbers [76] are commonly used to refer to genes, but it is important to note that they specifically classify enzymatic reactions. The relationship is not always a one-to-one mapping. For instance, multiple different enzymes can perform the same reaction, and a single gene might encode an enzyme that carries out several different reactions, each with its own E.C. number [76].

The Kyoto Encyclopedia of Genes and Genomes (KEGG) database [77] is widely used as a reference for gene, pathway and reaction mapping. The KO database contains molecular functions represented by highly specific orthologs with high granularity. A single KO includes only highly similar sequences; therefore, while multiple KOs can represent a single gene, it should not be expected for a single KO to represent multiple genes [77]. This rendered KOs perfect for this study, given the specificity that we want to achieve when identifying homologs of the same gene across multiple genomes and has been the reason for their use for functional characterization of *Klebsiella* strains [78,80].

#### Biomarker validation

To be effective SSMGs, the sequences of the KOs need to be as unique as possible, especially in amplicon rather than WGS. For each of the identified KO biomarkers, we utilized the ‘Function search’ in IMG/M with the ‘KEGG orthology ID list’ filter to locate all associated genes. These genes were individually selected and added to the genome cart. We then extracted protein sequences and used blastp to compare them against genomes of the other PQV species, retaining only hits with an *e*-value of 1e−5 or lower. For each gene, we calculated the minimum, maximum, mean and median percentage of sequence identity over shared coverage regions. This rigorous validation process ensured that our biomarkers’ presence or absence was thoroughly verified.

## Results and discussion

### Genome identification, selection and quality evaluation

We identified 470 genomes classified to PQV (as of 14 January 2025) either in the GTDB or the NCBI database according to the IMG/M database. The majority of these genomes were classified as *K. pneumoniae*, with 420 in the NCBI database and 413 in the GTDB. Due to the overrepresentation of *K. pneumoniae* genomes, it was necessary to use only a subset to ensure equal representation and comparability across species.

We selected all genomes classified as either *K. quasipneumoniae* or *K. variicola* in both the GTDB and NCBI databases (24 and 23 genomes, respectively), along with all genomes mismatching between the GTDB and the NCBI as designated in the IMG/M database (8 genomes, including 2 without a GTDB classification) (Table 1). To ensure parity, we randomly selected 23 genomes, equal to the lower count of the other two species, classified as *K. pneumoniae* in both the NCBI and GTDB (Table 1). Three *K. oxytoca* genomes were chosen to make up the outgroup for the two phylogenetic analyses, given the species’ phylogenetic distinctiveness from PQV [40]. This resulted in a final dataset of 81 genomes (Table S1, available in the online Supplementary Material).

**Table 1.** Distribution of genome classifications across the NCBI and GTDB databases as per the IMG/M web service, including the PQV genomes chosen for phylogenetic analysis and the three *K. oxytoca* genomes chosen as the outgroup

Classification		Count	Total count
Identical classification in both NCBI and GTDB	*K. oxytoca*	3	3
	*K. pneumoniae*	23	23
	*K. quasipneumoniae*	24	24
	*K. variicola*	23	23
Discrepancies in classification between NCBI → GTDB	*K. pneumoniae → K. quasipneumoniae*	2	8
	*K. pneumoniae → K. variicola*	3	
	*Kl. variicola → K. quasipneumoniae*	1	
	*K. pneumoniae → Unidentified in GTDB*	2	
**Total genomes**		**81**	

The six genomes with discrepant taxonomic classifications between the GTDB and NCBI represented 1.28% of the 470 PQV genomes. Given the clinical importance of accurately classifying these three species, this is a significantly high misclassification rate. As of 20 April 2025, even though the NCBI had adopted the GTDB classifications, this has yet to be propagated to the IMG/M database and, therefore, still presents a source of error. There is also no guarantee that such discrepancies would not arise in the future due to the differences in how taxonomic classifications are set for each database. While NCBI accepts user-submitted taxonomic classifications and updates them in real time, the GTDB uses a more systematic approach, updating its classifications in sync with Reference Sequence Database releases [34].

Before proceeding with phylogenetic analyses, we assessed the quality of all selected genomes. According to CheckM, all genomes were 99.48–100% complete (median of 100%) and had contamination ranging from 0.08 to 2.32% (median of 0.49%) (Table S2). These levels of contamination are well within the range expected of high-quality genomes, frequently reflecting gene duplication and/or HGT events [81,82]. Such events are known to drive XDR and hypervirulence in *Klebsiella* spp. [9]. Based on these quality metrics, all genomes selected here were deemed suitable for downstream analyses.

### Multimodal classification of PQV genomes

**Fig. 2.**
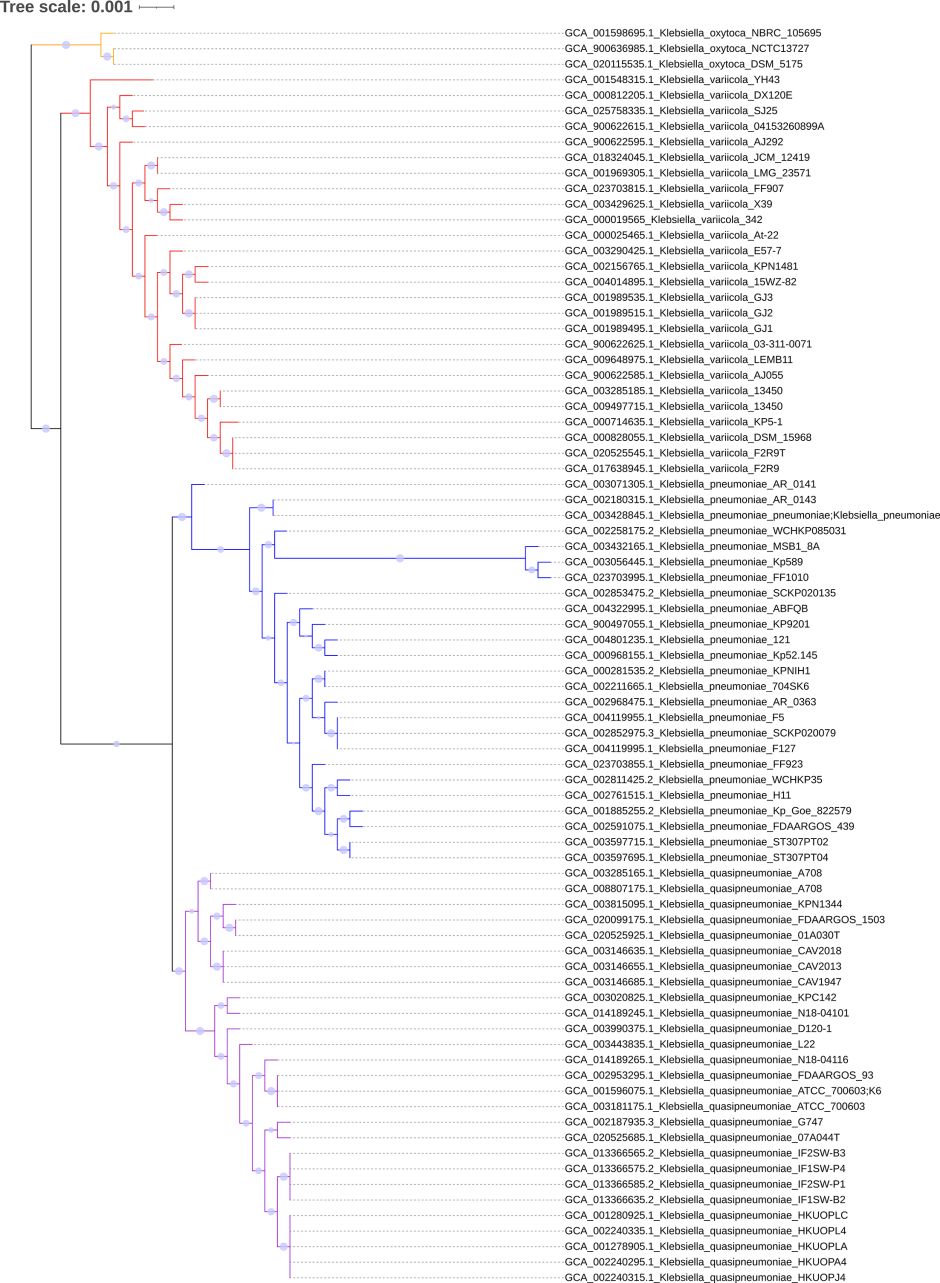
Rooted whole-genome phylogenetic tree of 81 *Klebsiella* genomes, showing clear species-level clustering. The genomes are grouped into four major clades corresponding to *K. oxytoca* (orange), *K. variicola* (red), *K. quasipneumoniae* (purple) and *K. pneumoniae* (blue). The clustering pattern is consistent with GTDB classifications.

The three *K. oxytoca* genomes were clustered together distinctly from the PQV genomes in the rooted whole-genome (multi-gene/concatenated protein) phylogenetic tree (Fig. 2). This aligns with the current knowledge of *Klebsiella* phylogenetics [40]. The 81 PQV genomes were split into 4 distinct clusters representing each of the 4 species, and excluding the 2 genomes that were not present in the GTDB at the time of analysis, the classifications were entirely concordant with the GTDB classifications. The GTDB-tk tool affirmed these classifications and further confirmed the remaining two genomes as *K. pneumoniae*, consistent with the whole-genome phylogenetic analysis (Fig. 2). It should be noted that with the Release 10-RS226 (16 April 2025) of the GTDB, the two previously unclassified genomes (GCA_004801235.1 and GCA_004322995.1) were incorporated in the database [34] with the correct taxonomy. This delayed addition does underscore the necessity for the GTDB-tk as a tool for accurate genome classifications in the interim of the updates.

In contrast, the 16S rRNA phylogenetic tree was highly inconsistent (Fig. S1). The *K. oxytoca* sequences were not even mostly clustered together and thus could not form an outgroup for accurate rooting of the tree. As an example, ‘2732826106 16S rRNA, Bacterial SSU *Klebsiella oxytoca* NBRC 105695’ was clustered exclusively with *K. variicola* sequences, distinct from all other *K. oxytoca* sequences. Similar inconsistencies also applied to PQV sequences (Fig. S2). Such inconsistencies are known issues with using the 16S rRNA gene for phylogenetic analyses of lower taxonomic ranks, unable to robustly delineate species or strains [51]. Thus, it is clear that the 16S rRNA gene sequence should not be used to phylogenetically classify PQV species and likely *Klebsiella* in general, given the irregular placement of the *K. oxytoca* sequences.

**Fig. 3.**
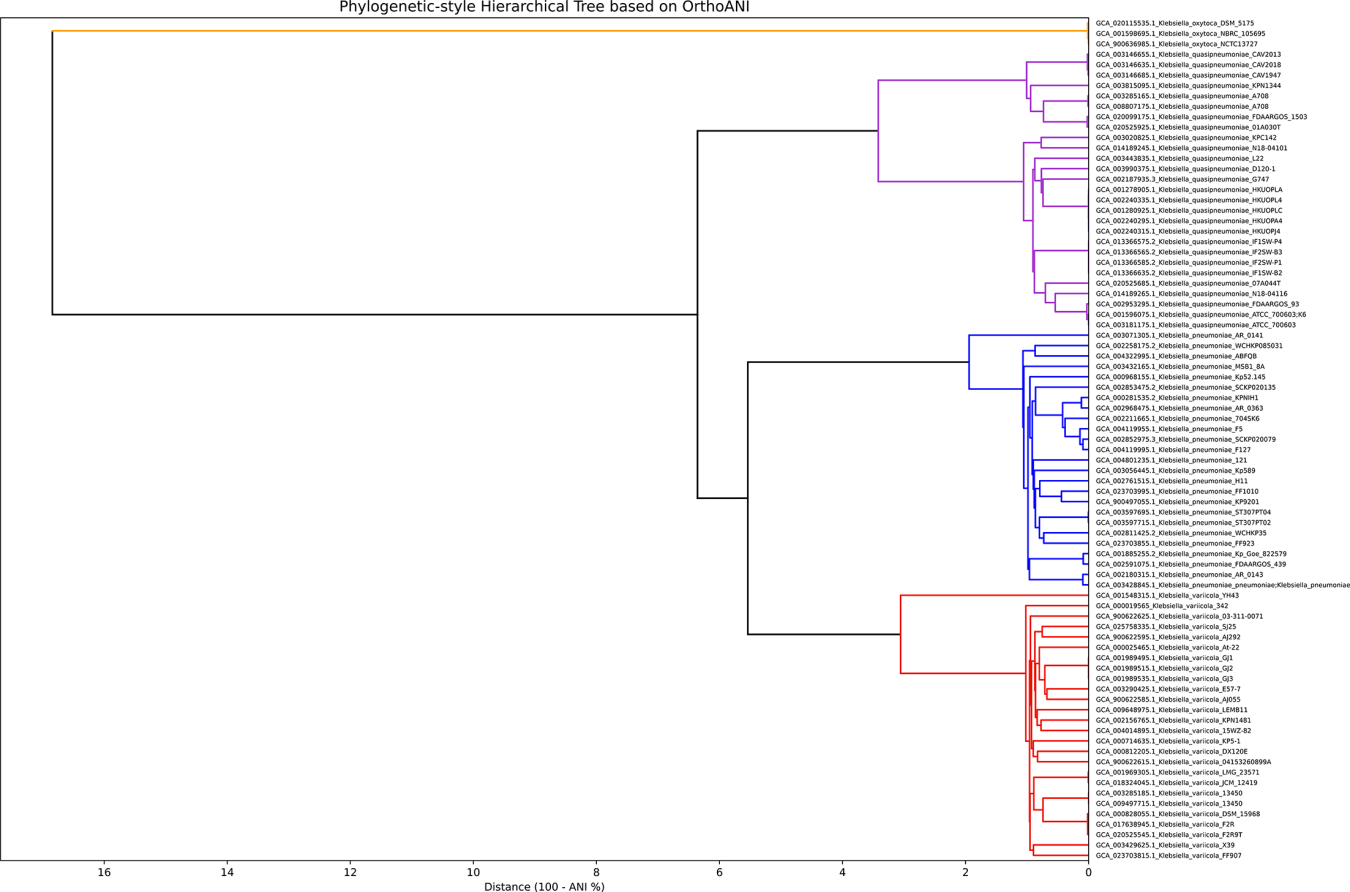
OrthoANI-based hierarchical clustering dendrogram of 81 *Klebsiella* genomes, showing 3 distinct genome clusters. The clusters correspond to *K. oxytoca* (orange), *K. variicola* (red), *K. quasipneumoniae* (purple) and *K. pneumoniae* (blue). The clustering is fully concordant with GTDB taxonomic classifications.

The OrthoANI hierarchical clustering dendrogram (Fig. 3) formed four distinct clusters, namely *K. pneumoniae*, *K. variicola*, *K. quasipneumoniae* and *K. oxytoca*. These corresponded with the clustering of the whole-genome phylogenetic tree (Fig. 2) at the species level. Specifically, *K. oxytoca* formed a distant outgroup with OrthoANI values of only ~83% compared to other clusters. This helped to confirm that it is a distinct species. *K. pneumoniae*, *K. variicola* and *K. quasipneumoniae* each showed internal OrthoANI values above 99%, but interspecies OrthoANI values between them ranged from ~93 to 95 % which supports their classification as distinct but closely related species.

**Fig. 4.**
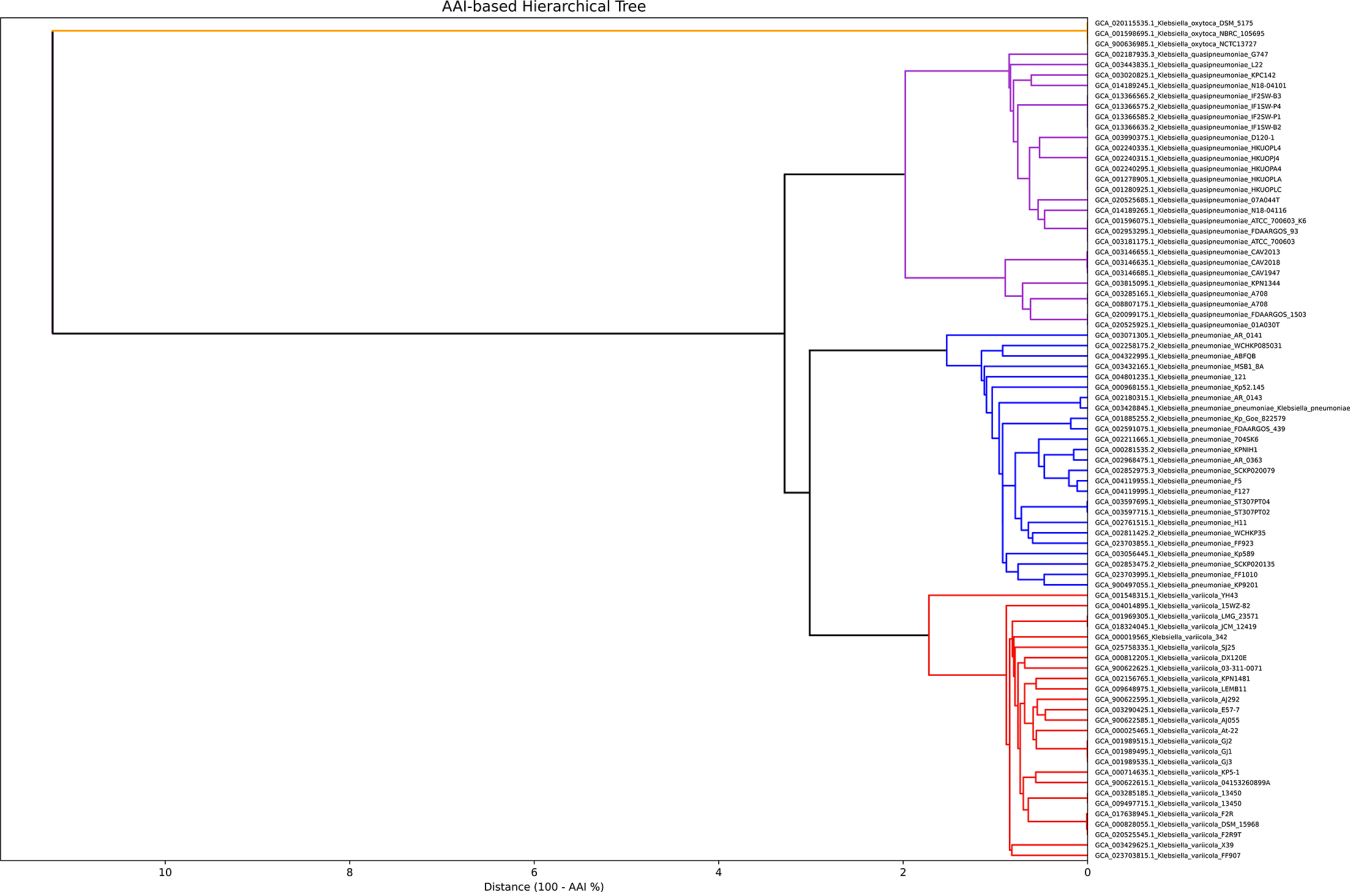
AAI-based hierarchical clustering dendrogram of 81 *Klebsiella* genomes, showing 3 distinct genome clusters. The clusters correspond to *K. oxytoca* (orange), *K. variicola* (red), *K. quasipneumoniae* (purple) and *K. pneumoniae* (blue). The clustering is fully concordant with GTDB taxonomic classifications.

The AAI-based dendrogram (Fig. 4) provided further resolution at the species level. Consistent with the OrthoANI clustering, four major clades were observed. *K. oxytoca* formed a well-separated outgroup, whereas *K. quasipneumoniae*, *K. pneumoniae* and *K. variicola* each formed tightly clustered, species-specific groups. Within each species cluster, the average AAI values exceeded 95%, the generally accepted threshold for species delineation, whereas interspecies AAI values fell between ~93 and 95 %. This pattern is congruent with the OrthoANI and phylogenomic clustering results.

**Fig. 5.**
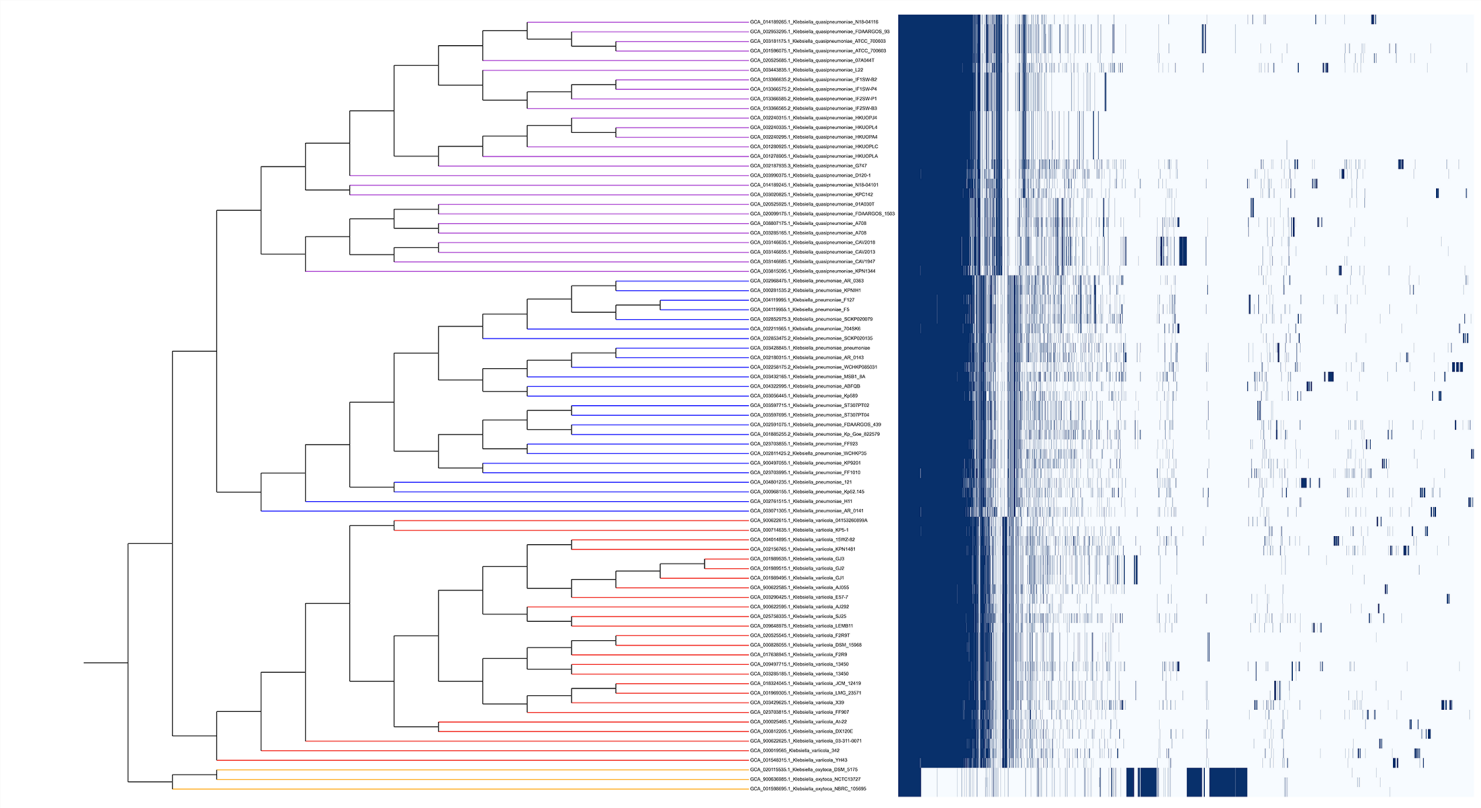
Pangenome matrix of *Klebsiella* genomes showing three distinct genome clusters corresponding to *K. oxytoca* (orange), *K. variicola* (red), *K. quasipneumoniae* (purple) and *K. pneumoniae* (blue). The dendrogram on the left, based on gene presence and absence patterns, clearly separates the genomes and aligns according to GTDB. The heatmap visualizes the gene content across all genomes, where dark blue indicates the presence of a gene cluster and white denotes its absence.

Similarly, the pangenome matrix (Fig. 5) showed distinct gene content patterns corresponding to the species clusterings we identified through phylogenetic analysis, OrthoANI and AAI-based dendrograms. *K. oxytoca* genomes consistently formed a distinct outgroup, separated from the PQV complex by their unique accessory genes. Within the PQV clade, *K. pneumoniae*, *K. variicola* and *K. quasipneumoniae* each formed tight, well-supported clusters corresponding to their GTDB classifications.

Thus, the six genomes with classification discrepancies between databases all had OrthoANI, AAI and pangenomic characteristics that aligned with their GTDB classifications rather than their original NCBI designations. The species-level congruency across whole-genome phylogenetic analysis, OrthoANI and AAI hierarchical clustering, and pangenomic analysis enforces the robustness of our species-delineation approach. Further, it proves the unreliability of using the 16S rRNA gene sequence as the basis for phylogenetic classification of PQV. The congruency also suggests that for the purposes of species delineation, relying on any one of the four methodologies (whole-genome phylogenetic analysis, OrthoANI and AAI hierarchical clustering and pangenomic analysis) is enough to accurately classify PQV genomes. Nonetheless, we recommend using all four methods to ensure maximum classification confidence, especially given they can be generated from the same data source. Based on our comprehensive analyses and the convergence of multiple methodologies, the final taxonomy of the 81 genomes included 25 *K*. *pneumoniae*, 27 *K*. *quasipneumoniae*, 26 *K*. *variicola* and 3 *K*. *oxytoca* (Table 2).

**Table 2.** Final taxonomic classifications according to the whole-genome phylogenetic analysis, OrthoANI hierarchical clustering, AAI hierarchical clustering and pangenomic analysis

Item	Count
*K. pneumoniae*	25
*K. quasipneumoniae*	27
*K. variicola*	26
*K. oxytoca*	3
**Total**	**81**

The GTDB’s robustness highlights this database and its companion classification tool, GTDB-tk, as the gold standard for taxonomic classification of PQV genomes. Conversely, even when the NCBI has adopted the GTDB classifications, the changes may take time to propagate to other databases, as seen with the IMG/M database in this study. Furthermore, as previously mentioned, unlike the NCBI database, taxonomic classification by the GTDB is systematic, preventing misclassifications introduced directly by user annotations. The GTDB-tk tool is publicly available via the KBase web platform [41]. Therefore, anyone with a bacterial (and archaeal) genome can freely classify it without needing to rely on local computational resources.

### Pangenomic analysis

**Fig. 6.**
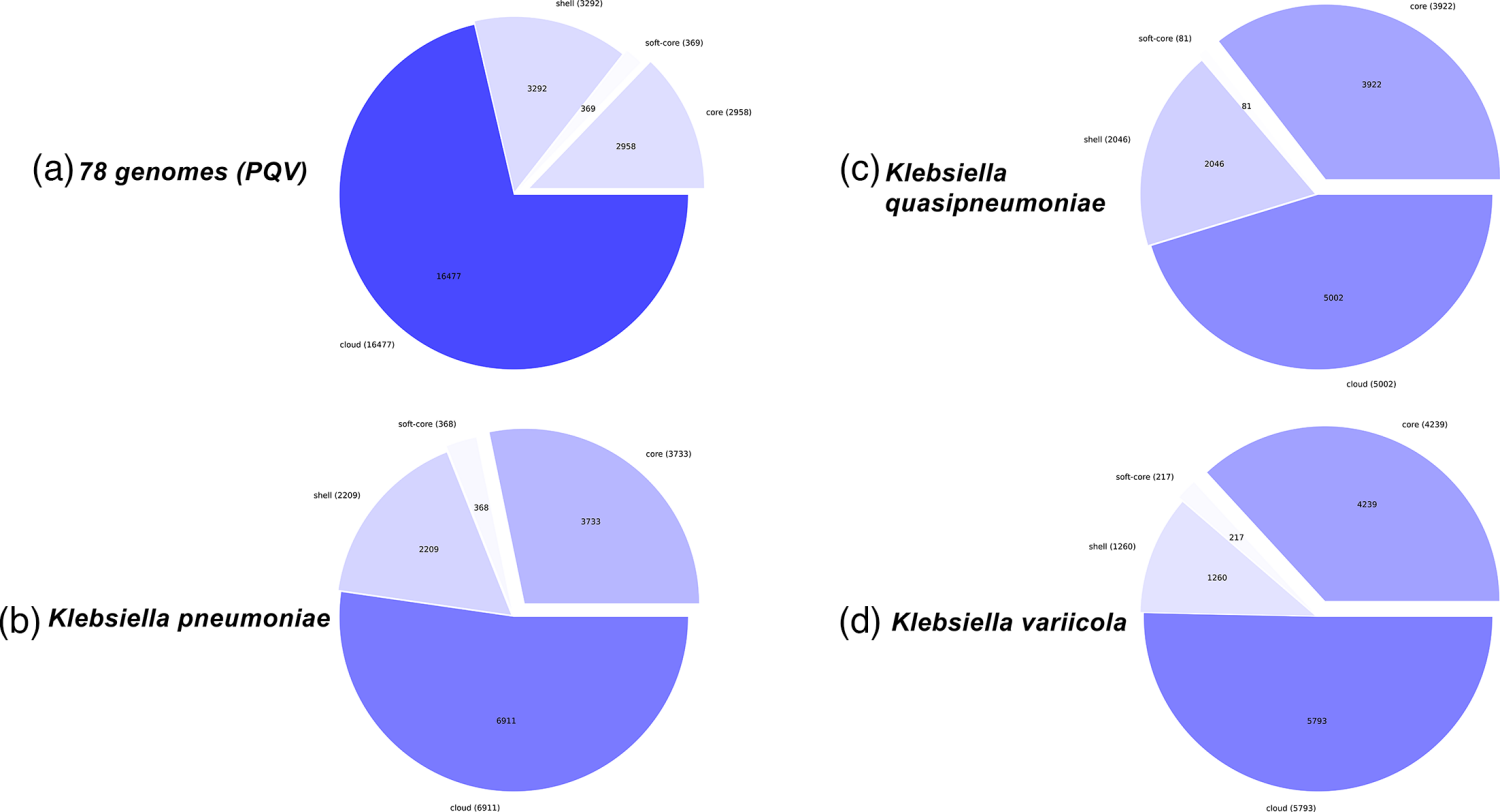
Pangenome composition pie charts for *Klebsiella* species. (**a**) All 78 PQV genomes combined. (**b**) *K. pneumoniae* genomes. (**c**) *K. quasipneumoniae* genomes. (**d**) *K. variicola* genomes. Each chart shows the distribution of core, soft-core, shell and cloud genes.

Pangenomic analysis found 23,096 gene clusters across all 78 PQV genomes used for species delineation (Fig. 6a). The prevalence of cloud genes (16,477) (71.3% of the total pangenome) across all species indicates an open pangenome, which is unsurprising given that *Klebsiella* species are known for their genetic plasticity and their constant acquisition of genetic material, often through HGT from other bacteria, plasmids or mobile genetic elements [9].

The 78 PQV genomes share 2,958 core genes (12.8% of the pangenome, present in at least 77 of the genomes). Examination at the species level of the genomes present in both the NCBI and GTDB databases (Table 1) found that *K. pneumoniae*, *K. quasipneumoniae* and *K. variicola* had 3,733, 3922, and 4239 core genes, respectively (Fig. 6b–d).

The higher number of core genes (4,240) (36.8% of its pangenome) indicates that *K. variicola* occupies a more specialized ecological niche than the other two species. Correspondingly, *K. pneumoniae* exhibits the most diverse pangenome, with 6,911 cloud genes (52.3%) cloud genes, aligning with its known adaptability and clinical significance. *K. quasipneumoniae* displays an intermediate pattern of genomic diversity. Indeed, *K. variicola,* despite its wide environmental associations, is found to comprise a range of core functions such as nitrogen fixation and plant growth promotion [83], in contrast to the highly diverse functions of *K. pneumoniae* (beyond its pathogenicity and related functions) [84].

Importantly, the sharp reduction in shared core genes when combining all species (2,958 genes) (only 12.8% of the pangenome) also shows the strong genomic divergence between PQV species. Although each species maintains a relatively large and stable core genome, most genes are unique to each species. This distinction provides a biological foundation for identifying SSMGs that are consistently present within a single species but absent in others, making them ideal for accurate species delineation. To ensure robust and comparable results, we selected an equal number of genomes for each species. We used 26 genomes per species, which was the maximum possible given the number of available *K. variicola* genomes.

### Species-specific marker gene identification using KEGG orthologies

We identified 22 KOs that were present in all genomes of one species but not in any genomes of the other 2 species or vice versa (Table 3). In particular, we identified 9, 13 and 17 KOs ubiquitously present in the genomes of *K. pneumoniae*, *K. variicola* and *K. quasipneumoniae*, respectively. This distribution does not correlate with the difference in core genes between the individual species and all three PQV species (Fig. 6a–d). There are multiple potential explanations for this discrepancy. First, we used a highly stringent 100% presence/absence criterion for SSMG identification, unlike the more relaxed definition used in the pangenome analysis. This filtered out many potential markers. Second, different ontologies are not congruent. For example, a single COG or pfam identifier can nonetheless include multiple KOs, and therefore, mapping is not expected to be one-to-one, thus resulting in count mismatches.

**Table 3.** Results of a blastp search of all gene sequences for each KO against genomes of species not containing the KO. Values are listed as percentage identity, regardless of actual coverage

KO ID	blastp versus											
	*K. pneumoniae*				*K. quasipneumoniae*				*K. variicola*			
	Min	Mean	Median	Max	Min	Mean	Median	Max	Min	Mean	Median	Max
K00055	–	–	–	–	22%	29%	29%	41%	–	–	–	–
K01130	–	–	–	–	–	–	–	–	24%	25%	25%	26%
K03430	–	–	–	–	–	–	–	–	23%	27%	27%	35%
K05306	–	–	–	–	–	–	–	–	0%	0%	0%	0%
K06146	21%	26%	25%	47%	21%	26%	25%	47%	–	–	–	–
K07507	–	–	–	–	–	–	–	–	0%	0%	0%	0%
K09955	0%	0%	0%	0%	–	–	–	–	0%	0%	0%	0%
K10018	19%	27%	29%	32%	–	–	–	–	–	–	–	–
K10019	26%	32%	33%	45%	–	–	–	–	–	–	–	–
K10020	24%	29%	29%	36%	–	–	–	–	–	–	–	–
K10021	20%	31%	30%	59%	–	–	–	–	–	–	–	–
K11081	–	–	–	–	–	–	–	–	23%	24%	25%	27%
K11082	–	–	–	–	–	–	–	–	22%	27%	27%	34%
K11083	–	–	–	–	–	–	–	–	25%	28%	27%	36%
K11084	–	–	–	–	–	–	–	–	21%	32%	32%	53%
K11616	27%	34%	38%	38%	–	–	–	–	–	–	–	–
K13794	19%	28%	28%	44%	–	–	–	–	–	–	–	–
K13795	0%	0%	0%	0%	–	–	–	–	–	–	–	–
K13796	22%	27%	26%	34%	–	–	–	–	–	–	–	–
K16958	19%	26%	26%	37%	19%	27%	26%	37%	–	–	–	–
K16959	22%	29%	29%	38%	22%	29%	29%	38%	–	–	–	–
K16960	19%	31%	31%	56%	21%	32%	31%	54%	–	–	–	–

Five of the KOs are potential positive SSMGs (i.e. the species is likely present if the marker is *present*)*,* and 17 are potential negative SSMGs (i.e. the species is likely present if the marker is *absent*) (Table 4). For positive SSMGs, four are specific to *K. variicola* and one to *K. quasipneumoniae* (Table 4). *K. pneumoniae* does not have any positive SSMGs; however, it may still be reliably identified using eight negative SSMGs. *K. quasipneumoniae* and *K. variicola* have one and eight negative SSMGs, respectively (Table 4).

**Table 4.** Number of genomes containing each of the KOs of interest and the total counts across all genomes. A single KO can map to multiple genes, which is the case whenever the gene count is higher than the genome count

KO ID	Gene name	KO definition	No. of genomes containing the KO			Total count	
			*K. pneumoniae*	*K. quasi* *-pneumoniae*	*K. variicola*	Total genome count	Total gene count
K00055		Aryl-alcohol dehydrogenase [EC:1.1.1.90]	26	0	26	52	54
K01130	*atsA, aslA*	Arylsulphatase [EC:3.1.6.1]	26	26	0	52	59
K03430	*phnW*	2-Aminoethylphosphonate-pyruvate transaminase [EC:2.6.1.37]	26	26	0	52	52
K05306	*phnX*	Phosphonoacetaldehyde hydrolase [EC:3.11.1.1]	26	26	0	52	52
K06146	*idnR, gntH*	LacI family transcriptional regulator, gluconate utilization system Gnt-II transcriptional activator	0	0	26	26	27
K07507	*mgtC*	Putative Mg²^+^ transporter-C (MgtC) family protein	26	26	0	52	52
K09955		Uncharacterized protein	0	26	0	26	26
K10018	*occT, nocT*	Octopine/nopaline transport system substrate-binding protein	0	26	26	52	52
K10019	*occM, nocM*	Octopine/nopaline transport system permease protein	0	26	26	52	52
K10020	*occQ, nocQ*	Octopine/nopaline transport system permease protein	0	26	26	52	52
K10021	*occP, nocP*	Octopine/nopaline transport system ATP-binding protein [EC:7.4.2.1]	0	26	26	52	52
K11081	*phnS*	2-Aminoethylphosphonate transport system substrate-binding protein	26	26	0	52	52
K11082	*phnV*	2-Aminoethylphosphonate transport system permease protein	26	26	0	52	52
K11083	*phnU*	2-Aminoethylphosphonate transport system permease protein	26	26	0	52	52
K11084	*phnT*	2-Aminoethylphosphonate transport system ATP-binding protein	26	26	0	52	52
K11616	*maeN*	Malate:Na+symporter	0	26	26	52	52
K13794	*tcuR*	LysR family transcriptional regulator, regulatory protein for tcuABC	0	26	26	52	52
K13795	*citB, tcuB*	Citrate/tricarballylate utilization protein	0	26	26	52	52
K13796	*cobZ, tcuA*	Tricarballylate dehydrogenase	0	26	26	52	52
K16958	*tcyL*	l-Cystine transport system permease protein	0	0	26	26	26
K16959	*tcyM*	l-Cystine transport system permease protein	0	0	26	26	26
K16960	*tcyN*	l-Cystine transport system ATP-binding protein [EC:7.4.2.1]	0	0	26	26	26

### Specificity validation of marker genes

For 18 of the 22 KOs, the blastp search revealed low coverage and identity matches with median identities of ≤38% (Table 3). Even distantly related genes can share some sequence identity due to conserved functional structures such as binding sites and anchor domains [85]. However, the minimal overlap observed here is evidence that sequences identified in other genomes represented either analogues or very distant paralogs. Regardless, these limited overlaps suggest that all 22 KOs identified are at least viable targets for WGS to delineate the PQV species accurately.

In contrast, each KO exhibited >90% identity within the same species. Combined with low interspecific coverage, there should also be ample opportunity for designing robust amplicon-based diagnostic assays [86]. This is true even for the four KOs with >50% identity matches (Table 3), whereby the dissimilarity is still significantly lower than expected for species of the same genus, where AAI is expected to be ≥65% [82]. All four KOs encode ATP-binding proteins for ATP-binding cassette (ABC) transporters (Table 4), and therefore, the higher identity is expected, reflecting the similarities needed for the ATP-binding domain [87]. Therefore, all 22 KOs can at this stage be classified as suitable SSMGs for the delineation of PQV.

Four KOs (K05306, K07507, K13795 and K09955) had no matches with the blastp search with an *e*-value cut-off of 1e−5, resulting in 0% identity (Table 3). This complete absence of homology in other species makes these ideal candidates for PCR or amplicon sequencing targets, with minimal risk of cross-reactivity. K05306 and K07507 are both negative markers for *K. variicola*, K13795 is a negative marker for *K. pneumoniae* and K09955 is a positive marker for *K. quasipneumoniae*. This means that using just these four SSMGs, or even a subset of three (using either K05306 or K07507), is enough to delineate the PQV species.

In WGS or full-length amplicon sequencing, each SSMG is expected to show a clear presence or absence in a genome. This reliability comes from two factors. First, the marker genes have very low blastp sequence coverage when compared to non-target species. Second, they also show low or no blastp identity to analogues or paralogs in other species.

They also show low or no blastp identity to analogues or paralogs in other species.

This is especially true for the four most specific SSMGs, which showed 0% identity to any non-target species in blastp analysis.

One important consequence of the low identity among these genetic elements, including the four SSMGs encoding ABC transporter ATP-binding proteins, is their potential to act as robust allelic variants. Even with traditional short-length amplicon sequencing, these variants may accurately delineate species, given that their potential identity is significantly lower than the typical >90% intraspecific identity.

### Metabolic pathways encoded by the species-specific marker genes

As the SSMGs were identified based on KO annotations, they are often associated with known biological functions. Examining these functions adds context to the genetic distinctions between PQV species. These metabolic associations of the SSMGs can translate into metabolic assays that can help delineate PQV in the laboratory setting.

Fourteen of the KOs represent genes encoding ABC transporters, specifically K10018-K10021 for octopine/nopaline [88], K11081-K11084 for 2-aminoethylphosphonate [89,90], K13794-K13796 for tricarballylate [91] and K16958-K16960 for l-cystine transport [92] (Table 4). K11616 encodes a malate:Na^+^ symporter [93], and K07507 encodes a putative Mg²^+^ transporter-C family protein [94]. K03430 and K05306 together are involved in 2-aminoethylphosphonate degradation [89], and K00055 and K01130 are involved in the metabolism of two distinct aryl compounds [95,96]. K06146 encodes a transcriptional regulator [97], and the activity of K09955 remains uncharacterized (Table 4).

While KOs involved in transport were common among the 22 identified, their distribution pattern varied (Table 4). K16958-K16960 were only found in *K. variicola* genomes; K10018-K10021, K11616 and K13794-K13796 were absent in *K. pneumoniae* genomes, while K11081-K11084 and K07507 were absent in *K. variicola* genomes. Notably, K03430 and K05306 were also absent from *K. variicola* genomes (Table 4). These two KOs and K11081-K11084 form a complete set of genes encoding the uptake and degradation of 2-aminoethylphosphonate [89,90]. 2-Aminoethylphosphonate belongs to the phosphonate class of compounds, which contain a C-P bond that is particularly difficult to break [98]. Only a few enzymes can break the bond, including phosphonoacetaldehyde hydrolase [99], i.e. K05306 in this study, allowing for the release of phosphate. The capability to degrade 2-aminoethylphosphonate fully, therefore, may give *K. pneumoniae* and *K. quasipneumoniae* a competitive advantage to leverage a ubiquitous phosphorus source that is not available to other micro-organisms.

For example, 2-aminoethylphosphonate is likely metabolized by *K. pneumoniae* and *K. quasipneumoniae*, but not by *K. variicola*, as aforementioned. However, further research is needed to confirm these potential delineating metabolic profiles. For example, SSMGs encoding the octopine/nopaline ABC transport system were identified for *K. quasipneumoniae* and *K. variicola* (Table 4). It is unclear which of octopine and/or nopaline can actually be transported, and while transport is indicative of catabolic capabilities, this is not definitive. Further laboratory research is needed to validate the potential of SSMG classification in distinguishing metabolic profiles. Nonetheless, this approach holds promise for additional beneficial applications.

### Increased robustness with gene sets

Many metabolic pathways are conserved and consist of specific genes that are gained and lost together, as they are required for complete end-to-end metabolism of particular substrates [100]. The loss of a single gene can cause the entire process to cease or cause a buildup of metabolites that directly or indirectly harm the organism. Therefore, genes involved in the same metabolic pathway, or more specifically, forming a single enzymatic complex or transport system, are generally retained unless the function is no longer beneficial to the organism [101].

Here, we have four sets of such genes, representing the four ABC transport systems as previously discussed (Table 4), one of which also includes two genes for downstream metabolism. To evaluate the degree of linkage within these gene sets, we identified them within all test genomes and determined whether they co-occurred within operons or as separate loci. In all cases, the genes encoding the octopine/nopaline transport system were found within a single operon, specifically in *occ/nocTQMP* order, which is different from both the originally described *occQMPT* and *nocPTQM* orders identified for *Agrobacterium tumefaciens* [102]. The three tricarballylate transport SSMGs were also found within the same operon, alongside *tcuC*, forming the *tcuRABC* gene cluster. Here, the order of genes is indeed highly typical of a wide range of bacteria [103,106], indicating well-known and consistent function. Interestingly, *tcuC* was also often detected in *K. pneumoniae*, though frequently as a single copy and without *tcuABC*. TcuC alone is enough for the import of tricarballylate, but is not involved in its metabolism [91]. In contrast, *K. variicola* and *K. quasipneumoniae* typically carried two copies – one as part of the tricarballylate transport operon, the other distinct. Thus, the presence of *tcuC* in *K. pneumoniae* does not necessarily indicate a loss of other *tcu* genes; rather, it may have been independently acquired and serve a different function. Similarly, *tcyNLMK* is also found together in the same operon in all *K. variicola* genomes, with *tcyK* also identified in *K. quasipneumoniae* genomes. The *tcyK* genes found in the *K. quasipneumoniae* genomes were flanked by transposable elements and likely were independently acquired via HGT. Lastly, *phnSTUV* are found together, preceded by *phnR* encoding the phosphonate utilization transcriptional regulator. *phnWX* are also located in the genomic region, flanking *phnR*, but on the opposite strand.

It is clear then that genes from each set are co-located and comprise functional operons, co-acquired and likely resistant to single-gene losses. These sets can allow identification processes to be even more robust, as the absence of one or more genes is unlikely if the others are successfully sequenced. This can be particularly effective with shotgun metagenomic sequencing, where there can be a higher likelihood of gene fragments being assembled. The importance of analysing genes as functional sets operating within biological pathways, rather than individually, can be observed through metabolic differentiation.

### Species-specific marker gene panel recommendation

While the use of all 22 SSMGs optimizes the robustness of delineating PQV, it is not always possible to include them all in a single panel. Due to budget or even technological constraints, a minimal panel may only be possible. In that case, we would recommend K13795, K09955 and either K05306 or K07507 as the minimum three SSMGs to delineate PQV. If the goal is to differentiate between only two species, a single SSMG can be sufficient. However, due to the close relatedness of the PQV complex, it is advisable to always rely on a three-SSMG panel as the minimum standard. K05306 is preferable to K07507 given the former’s involvement in 2-aminoethylphosphonate, which is likely to be core to both *K. pneumoniae* and *K. quasipneumoniae*.

The SSMGs that are part of these gene sets are effective whether they are used together or on their own. However, some additional care may be needed for the SSMGs encoding ATP-binding proteins due to potential sequence conservation across species. Conversely, the genes encoding substrate-binding proteins, i.e. K10018, K11081 and K13795 (Table 4), are preferable given their substrate specificity translates to sequence uniqueness. Indeed, these SSMGs have lower blastp identities to potential analogues/paralogs (Table 4), with K13795 among those with zero matches. K01130 and K11616 are the least preferred SSMGs, as they do not belong to gene sets nor have>0% blastp matches. Moreover, their species-differentiating functions are already covered by more reliable SSMGs in the panel. Nonetheless, it should still be reiterated that, based on uniqueness validation, all 22 SSMGs can be viable biomarkers for delineating PQV species.

**The capability of SSMGs to distinguish PQV from other** ***Klebsiella*** **species**

While not directly within the scope of our research, given the clinical interest in delineating specific PQV species from each other, it can also be able to distinguish PQV species from other members of the genus. To evaluate if the 22 SSMGs found in this study could be potentially used as biomarkers for broader genus-wide distinctions, we evaluated their presence in all other *Klebsiella* genomes within the IMG/M database.

A total of 76 genomes were found that were classified as *Klebsiella* according to the GTDB. These represented 13 species, specifically *Klebsiella aerogenes*, *Klebsiella africana*, *Klebsiella grimontii*, *Klebsiella huaxiensis*, *Klebsiella michiganensis*, *K. oxytoca*, *Klebsiella quasivariicola*, *Klebsiella sp013705725* (placeholder GTDB name), *Klebsiella electrica*, *Klebsiella ornithinolytica*, *Klebsiella planticola* and *Klebsiella terrigena* (Table S3). The latter four species were previously classified in the genus *Raoultella* but were reunified with *Klebsiella* in 2021 [107].

No single SSMG can distinguish between one of the three PQV species and all 13 other *Klebsiella* species examined, although some do come close. For *K. pneumoniae* and *K. quasipneumoniae*, K06146 is a negative marker that is also only completely absent in *K. aerogenes*, *K. oxytoca_C* and some *K. oxytoca* genomes (Table S3). This can potentially help distinguish *K. pneumoniae* from ten of the additional *Klebsiella* species examined. Notably, the absence/presence profile of SSMGs for *K. variicola* differs significantly from that of the 13 additional *Klebsiella* species examined, with a total of 58.7% of cases. In contrast, the number of differences in absence/presence profiles between *K. pneumoniae* and *K. quasipneumoniae* compared to the additional 13 species was only 36.7 and 38.8 %, respectively.

Indeed, just K00055 or K01130 and any one of K03430, K05306, K10021, K11081-K11084 or K16958-K16960 could potentially differentiate between *K. variicola* and 12 of the 13 additional species, except either *K. electrica* or *K. grimontii*. A panel of 3 SSMGs could be enough to distinguish *K. variicola* from all 13 species. The potential different combinations of SSMGs to delineate either *K. pneumoniae* or *K. quasipneumoniae* from the 13 species are more limited. However, just two SSMGs can be enough. For *K. pneumoniae*, it would be a combination of K06146 and K13794. For *K. quasipneumoniae*, it would be K06146 and one of K10018-K10021 or K11616.

There are certain limitations here that should be noted. As previously stated, not all *Klebsiella* species could be investigated here, as not all *Klebsiella* genomes were available in the IMG/M database or were of high enough quality. For example, according to the GTDB, genomes exist for 26 *Klebsiella* species [34]. Additionally, this does not count species that lack sequenced genomes. Even among the additional 13 species investigated, 8 only have ≤5 representative genomes. *K. aerogenes* and *K. michiganensis* had the highest number of high-quality genomes, with 17 and 18, respectively. Yet, this is still relatively low compared to at least 26 for the PQV species. Therefore, the capability of the 22 SSMGs identified in this study to delineate between the PQV species is certain, but their broader applicability to distinguish the PQV species from other *Klebsiella* requires further research. Nonetheless, the insights here should provide a solid basis for future studies.

Importantly, the presence/absence of the sets of transporter genes is highly consistent intragenomically across all *Klebsiella* genomes examined beyond just the PQV, which further highlights the robustness of using gene sets for accurate taxonomic classifications.

## Conclusion

There are two major issues with the accurate taxonomic classification of *K. pneumoniae*, *K. quasipneumoniae* and *K. variicola*: inconsistent classifications across databases and inadequate rapid detection methods for these species. Misidentification of these species can lead to serious clinical consequences. To address this, we first established GTDB as a gold standard, demonstrating 100% concordance with our multimodal phylogenetic analyses for the genomes used in this study. This framework can be supported by using the GTDB-tk tool for rapid classification, as well as other methods like whole-genome phylogenetics, OrthoANI and AAI clustering and pangenomic analysis for more in-depth verification of unclassified genomes.

With a reliable phylogenetic framework established, we identified 22 SSMGs that consistently distinguish between the three PQV species that consistently distinguish between the three *PQV* species. This offers a practical alternative to whole-genome approaches for routine identification. The presence/absence nature of these markers reduces the risk of errors due to mutations or sequencing discrepancies compared to allelic variant biomarkers. Additionally, they avoid the inaccuracies of phenotypic methods that can be influenced by variable transcriptional activities. Four SSMGs proved exceptionally robust, as they had no matches that could be identified with a blastp search with our specific parameters. We recommend using a three-gene panel using K13795, K09955 and either K05306 or K07507. However, all 22 identified SSMGs demonstrate strong reliability. This allows users the flexibility to select markers and design assays that best fit their specific requirements.

## Supplementary material

10.1099/jmm.0.002102 Uncited Supplementary Material 1.

10.1099/jmm.0.002102 Uncited Table S1.

## Abbreviations

AAI: average amino acid identity
ABC: ATP-binding cassette
ANI: average nucleotide identity
blastp: Basic Local Alignment Search Tool for proteins
COG: Clusters of Orthologous Genes
E.C.: enzyme commission
GTDB-tk: Genome Taxonomy Database Toolkit
HGT: horizontal gene transfer
IMG/M: Integrated Microbial Genomes and Microbiomes
KBase: Department of Energy Systems Biology Knowledgebase
KEGG: Kyoto Encyclopedia of Genes and Genomes
KO: KEGG Orthology
KpSC: *Klebsiella pneumoniae* species complex
NCBI: National Center for Biotechnology Information
OrthoANI: orthologous average nucleotide identity
PQV: *Klebsiella pneumoniae*–*quasipneumoniae*–*variicola*
SSMGs: species-specific marker genes
WGS: whole-genome sequencing
XDR: extensively drug-resistant
