# Supplementary material for: Identification of robust species-specific marker genes for the demarcation of the Klebsiella pneumoniae–quasipneumoniae–variicola complex
Source: J Med Microbiol. 2025 Nov 17;74(11):002102. doi: 10.1099/jmm.0.002102 (PMC12623006; doi:10.1099/jmm.0.002102)
Supplement: Uncited Supplementary Material 1. [file jmm-74-02102-s001.pdf]

Supplementary tables

Supplementary Table S1. List of the 81 genomes used in the phylogenetic analysis. These included PQV genomes and three *Klebsiella oxytoca* genomes to make up the outgroup. Species classifications are per the IMG/M database [1].

NCBI Assembly Accession	NCBI Species	GTDB Species
GCA_001598695.1	*Klebsiella oxytoca*	*Klebsiella oxytoca*
GCA_900636985.1	*Klebsiella oxytoca*	*Klebsiella oxytoca*
GCA_020115535.1	*Klebsiella oxytoca*	*Klebsiella oxytoca*
GCA_000968155.1	*Klebsiella pneumoniae*	*Klebsiella pneumoniae*
GCA_001885255.2	*Klebsiella pneumoniae*	*Klebsiella pneumoniae*
GCA_002968475.1	*Klebsiella pneumoniae*	*Klebsiella pneumoniae*
GCA_002591075.1	*Klebsiella pneumoniae*	*Klebsiella pneumoniae*
GCA_900497055.1	*Klebsiella pneumoniae*	*Klebsiella pneumoniae*
GCA_003071305.1	*Klebsiella pneumoniae*	*Klebsiella pneumoniae*
GCA_003597695.1	*Klebsiella pneumoniae*	*Klebsiella pneumoniae*
GCA_002180315.1	*Klebsiella pneumoniae*	*Klebsiella pneumoniae*
GCA_002211665.1	*Klebsiella pneumoniae*	*Klebsiella pneumoniae*
GCA_003432165.1	*Klebsiella pneumoniae*	*Klebsiella pneumoniae*
GCA_003056445.1	*Klebsiella pneumoniae*	*Klebsiella pneumoniae*
GCA_004119955.1	*Klebsiella pneumoniae*	*Klebsiella pneumoniae*
GCA_003428845.1	*Klebsiella pneumoniae*	*Klebsiella pneumoniae*
GCA_004119995.1	*Klebsiella pneumoniae*	*Klebsiella pneumoniae*
GCA_002258175.2	*Klebsiella pneumoniae*	*Klebsiella pneumoniae*
GCA_002761515.1	*Klebsiella pneumoniae*	*Klebsiella pneumoniae*
GCA_002852975.3	*Klebsiella pneumoniae*	*Klebsiella pneumoniae*
GCA_002811425.2	*Klebsiella pneumoniae*	*Klebsiella pneumoniae*
GCA_000281535.2	*Klebsiella pneumoniae*	*Klebsiella pneumoniae*
GCA_003597715.1	*Klebsiella pneumoniae*	*Klebsiella pneumoniae*
GCA_023703855.1	*Klebsiella pneumoniae*	*Klebsiella pneumoniae*
GCA_023703995.1	*Klebsiella pneumoniae*	*Klebsiella pneumoniae*
GCA_002853475.2	*Klebsiella pneumoniae*	*Klebsiella pneumoniae*
GCA_004801235.1	*Klebsiella pneumoniae*	Unidentified
GCA_004322995.1	*Klebsiella pneumoniae*	Unidentified
GCA_001596075.1	*Klebsiella quasipneumoniae*	*Klebsiella quasipneumoniae*

GCA_001280925.1	*Klebsiella pneumoniae*	*Klebsiella quasipneumoniae*
GCA_001278905.1	*Klebsiella variicola*	*Klebsiella quasipneumoniae*
GCA_003146685.1	*Klebsiella quasipneumoniae*	*Klebsiella quasipneumoniae*
GCA_003443835.1	*Klebsiella quasipneumoniae*	*Klebsiella quasipneumoniae*
GCA_002240295.1	*Klebsiella quasipneumoniae*	*Klebsiella quasipneumoniae*
GCA_003146635.1	*Klebsiella quasipneumoniae*	*Klebsiella quasipneumoniae*
GCA_003815095.1	*Klebsiella pneumoniae*	*Klebsiella quasipneumoniae*
GCA_003285165.1	*Klebsiella quasipneumoniae*	*Klebsiella quasipneumoniae*
GCA_014189245.1	*Klebsiella quasipneumoniae*	*Klebsiella quasipneumoniae*
GCA_003990375.1	*Klebsiella quasipneumoniae*	*Klebsiella quasipneumoniae*
GCA_014189265.1	*Klebsiella quasipneumoniae*	*Klebsiella quasipneumoniae*
GCA_003181175.1	*Klebsiella quasipneumoniae*	*Klebsiella quasipneumoniae*
GCA_002953295.1	*Klebsiella quasipneumoniae*	*Klebsiella quasipneumoniae*
GCA_002187935.3	*Klebsiella quasipneumoniae*	*Klebsiella quasipneumoniae*
GCA_003020825.1	*Klebsiella quasipneumoniae*	*Klebsiella quasipneumoniae*
GCA_008807175.1	*Klebsiella quasipneumoniae*	*Klebsiella quasipneumoniae*
GCA_002240315.1	*Klebsiella quasipneumoniae*	*Klebsiella quasipneumoniae*
GCA_002240335.1	*Klebsiella quasipneumoniae*	*Klebsiella quasipneumoniae*
GCA_013366575.2	*Klebsiella quasipneumoniae*	*Klebsiella quasipneumoniae*
GCA_013366585.2	*Klebsiella quasipneumoniae*	*Klebsiella quasipneumoniae*
GCA_013366565.2	*Klebsiella quasipneumoniae*	*Klebsiella quasipneumoniae*
GCA_013366635.2	*Klebsiella quasipneumoniae*	*Klebsiella quasipneumoniae*
GCA_003146655.1	*Klebsiella quasipneumoniae*	*Klebsiella quasipneumoniae*
GCA_020525685.1	*Klebsiella quasipneumoniae*	*Klebsiella quasipneumoniae*
GCA_020525925.1	*Klebsiella quasipneumoniae*	*Klebsiella quasipneumoniae*
GCA_020099175.1	*Klebsiella quasipneumoniae*	*Klebsiella quasipneumoniae*
GCA_000019565	*Klebsiella variicola*	*Klebsiella variicola*
GCA_000025465.1	*Klebsiella variicola*	*Klebsiella variicola*
GCA_000714635.1	*Klebsiella pneumoniae*	*Klebsiella variicola*
GCA_000828055.1	*Klebsiella variicola*	*Klebsiella variicola*
GCA_000812205.1	*Klebsiella variicola*	*Klebsiella variicola*
GCA_001548315.1	*Klebsiella pneumoniae*	*Klebsiella variicola*
GCA_001989515.1	*Klebsiella variicola*	*Klebsiella variicola*
GCA_900622595.1	*Klebsiella variicola*	*Klebsiella variicola*
GCA_900622615.1	*Klebsiella variicola*	*Klebsiella variicola*

GCA_900622625.1	*Klebsiella variicola*	*Klebsiella variicola*
GCA_003285185.1	*Klebsiella variicola*	*Klebsiella variicola*
GCA_001989535.1	*Klebsiella variicola*	*Klebsiella variicola*
GCA_900622585.1	*Klebsiella variicola*	*Klebsiella variicola*
GCA_001969305.1	*Klebsiella variicola*	*Klebsiella variicola*
GCA_003429625.1	*Klebsiella variicola*	*Klebsiella variicola*
GCA_001989495.1	*Klebsiella variicola*	*Klebsiella variicola*
GCA_003290425.1	*Klebsiella variicola*	*Klebsiella variicola*
GCA_004014895.1	*Klebsiella variicola*	*Klebsiella variicola*
GCA_002156765.1	*Klebsiella variicola*	*Klebsiella variicola*
GCA_009497715.1	*Klebsiella variicola*	*Klebsiella variicola*
GCA_009648975.1	*Klebsiella variicola*	*Klebsiella variicola*
GCA_023703815.1	*Klebsiella variicola*	*Klebsiella variicola*
GCA_025758335.1	*Klebsiella pneumoniae*	*Klebsiella variicola*
GCA_020525545.1	*Klebsiella variicola*	*Klebsiella variicola*
GCA_018324045.1	*Klebsiella variicola*	*Klebsiella variicola*
GCA_017638945.1	*Klebsiella variicola*	*Klebsiella variicola*

Supplementary Table S2. Genome completeness and contamination metrics as per CheckM v1.0.18 [2] for the *Klebsiella* genomes used for all phylogenetic analyses. Accession ID is that of GenBank. In every case, there were 1,005 lineage-specific comprising 324 marker sets assessed. 0 to 5+ counts are the number of duplicates. Both completeness and contamination in terms of percentages.

Accession ID	0	1	2	3	4	5+	Completeness (%)	Contamination (%)
GCA_000019565	0	999	6	0	0	0	100	0.49
GCA_000025465.1	0	998	7	0	0	0	100	0.78
GCA_000281535.2	0	1004	1	0	0	0	100	0.08
GCA_000714635.1	5	995	5	0	0	0	99.6	0.34
GCA_000812205.1	0	999	6	0	0	0	100	0.37
GCA_000828055.1	0	998	7	0	0	0	100	0.77
GCA_000968155.1	1	993	11	0	0	0	99.96	1.33
GCA_001278905.1	1	1000	4	0	0	0	99.99	0.39
GCA_001280925.1	1	1000	4	0	0	0	99.99	0.39
GCA_001548315.1	0	1001	4	0	0	0	100	0.32
GCA_001596075.1	0	1000	5	0	0	0	100	0.45
GCA_001598695.1	0	1001	4	0	0	0	100	0.21
GCA_001885255.2	0	999	6	0	0	0	100	0.8
GCA_001969305.1	0	998	7	0	0	0	100	0.64
GCA_001989495.1	0	1000	5	0	0	0	100	0.37
GCA_001989515.1	0	1000	5	0	0	0	100	0.37
GCA_001989535.1	0	1000	5	0	0	0	100	0.37
GCA_002156765.1	0	984	21	0	0	0	100	2.32
GCA_002180315.1	0	1002	3	0	0	0	100	0.28
GCA_002187935.3	0	996	9	0	0	0	100	1.16
GCA_002211665.1	0	998	7	0	0	0	100	0.74
GCA_002240295.1	1	1000	4	0	0	0	99.99	0.39
GCA_002240315.1	1	1000	4	0	0	0	99.99	0.39
GCA_002240335.1	1	1000	4	0	0	0	99.99	0.39
GCA_002258175.2	0	1000	5	0	0	0	100	0.56
GCA_002591075.1	0	997	8	0	0	0	100	0.73
GCA_002761515.1	0	1002	3	0	0	0	100	0.46
GCA_002811425.2	0	1002	3	0	0	0	100	0.39
GCA_002852975.3	0	997	8	0	0	0	100	0.71
GCA_002853475.2	0	1000	5	0	0	0	100	0.59
GCA_002953295.1	0	1001	4	0	0	0	100	0.39
GCA_002968475.1	0	997	8	0	0	0	100	0.45
GCA_003020825.1	1	999	5	0	0	0	99.99	0.72
GCA_003056445.1	0	990	15	0	0	0	100	1.62
GCA_003071305.1	0	999	6	0	0	0	100	0.85
GCA_003146635.1	0	994	10	1	0	0	100	1.58

GCA_003146655.1	0	994	10	1	0	0	100	1.58
GCA_003146685.1	0	994	10	1	0	0	100	1.58
GCA_003181175.1	0	1001	4	0	0	0	100	0.39
GCA_003285165.1	1	1002	2	0	0	0	99.99	0.18
GCA_003285185.1	0	1000	5	0	0	0	100	0.63
GCA_003290425.1	1	998	6	0	0	0	99.9	0.63
GCA_003428845.1	0	1000	5	0	0	0	100	0.33
GCA_003429625.1	1	994	10	0	0	0	99.94	0.73
GCA_003432165.1	0	990	15	0	0	0	100	1.41
GCA_003443835.1	0	993	12	0	0	0	100	1.07
GCA_003597695.1	0	1003	2	0	0	0	100	0.39
GCA_003597715.1	0	1003	2	0	0	0	100	0.39
GCA_003815095.1	1	1002	2	0	0	0	99.99	0.18
GCA_003990375.1	0	1000	5	0	0	0	100	0.69
GCA_004014895.1	0	995	10	0	0	0	100	0.96
GCA_004119955.1	0	995	10	0	0	0	100	1.1
GCA_004119995.1	0	1001	4	0	0	0	100	0.54
GCA_004322995.1	0	1000	5	0	0	0	100	0.33
GCA_004801235.1	0	999	6	0	0	0	100	0.5
GCA_008807175.1	0	1002	3	0	0	0	100	0.22
GCA_009497715.1	0	995	10	0	0	0	100	1.19
GCA_009648975.1	0	1000	5	0	0	0	100	0.63
GCA_013366565.2	1	1001	3	0	0	0	99.99	0.33
GCA_013366575.2	1	1001	3	0	0	0	99.99	0.33
GCA_013366585.2	1	1001	3	0	0	0	99.99	0.33
GCA_013366635.2	1	1001	3	0	0	0	99.99	0.33
GCA_014189245.1	1	999	5	0	0	0	99.96	0.53
GCA_014189265.1	1	996	8	0	0	0	99.99	0.58
GCA_017638945.1	1	997	7	0	0	0	99.92	0.77
GCA_018324045.1	0	998	7	0	0	0	100	0.64
GCA_020099175.1	1	997	7	0	0	0	99.99	0.95
GCA_020115535.1	1	1000	4	0	0	0	99.69	0.21
GCA_020525545.1	0	998	7	0	0	0	100	0.77
GCA_020525685.1	1	1000	4	0	0	0	99.99	0.39
GCA_020525925.1	1	997	7	0	0	0	99.99	0.95
GCA_023703815.1	0	1003	2	0	0	0	100	0.18
GCA_023703855.1	1	1003	1	0	0	0	99.92	0.08
GCA_023703995.1	7	991	7	0	0	0	99.48	0.61
GCA_025758335.1	0	1001	4	0	0	0	100	0.32
GCA_900497055.1	0	998	7	0	0	0	100	0.79
GCA_900622585.1	0	1000	5	0	0	0	100	0.48

GCA_900622595.1	0	998	7	0	0	0	100	0.94
GCA_900622615.1	0	1001	4	0	0	0	100	0.32
GCA_900622625.1	0	1001	4	0	0	0	100	0.32
GCA_900636985.1	0	1001	4	0	0	0	100	0.21

Supplementary Table S3. Counts for the SSMGs identified for PQV in other *Klebsiella* genomes extracted from the IMG/M database.

[Supplementary Table S3 is included as a separate file]

Supplementary figures

[Supplementary Figure S1 is included as a separate pdf to retain resolution]

Supplementary Figure S1: Phylogenetic analysis of the 16S genes found in the 78 PQV genomes used in this study, along with three *Klebsiella oxytoca* genomes that were meant to form an outgroup. Labels were IMG/M gene IDs parsed directly from the database.

References

1. **Chen I-MA, Chu K, Palaniappan K, Ratner A, Huang J, *et al.*** The IMG/M data management and analysis system v.7: content updates and new features. *Nucleic Acids Res* 2023;51:D723–D732.

2. **Parks DH, Imelfort M, Skennerton CT, Hugenholtz P, Tyson GW**. CheckM: assessing the quality of microbial genomes recovered from isolates, single cells, and metagenomes. *Genome Res* 2015;25:1043–1055.

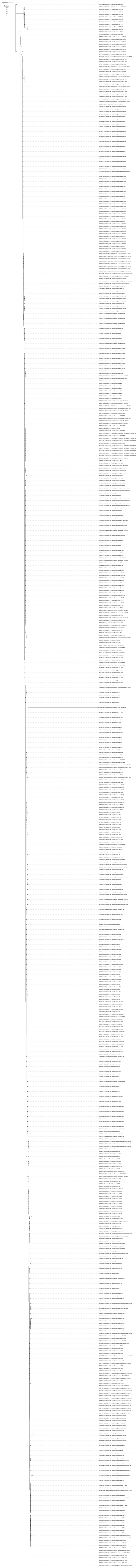
Tree scale: 0.01
bootstrap
0.73
0.73
0.73
0.73
0.73
8078245158 16S ribosomal RNA Klebsiella pneumoniae FF1010
2732824631 16S rRNA. Bacterial SSU Klebsiella oxytoca NBRC 105695
8117969432 16S ribosomal RNA Klebsiella oxytoca NCTC 13727
8117965427 16S ribosomal RNA Klebsiella oxytoca NCTC 13727
8117964924 16S ribosomal RNA Klebsiella oxytoca NCTC 13727
8117966321 16S ribosomal RNA Klebsiella oxytoca NCTC 13727
8117969837 16S ribosomal RNA Klebsiella oxytoca NCTC 13727
8117964803 16S ribosomal RNA Klebsiella oxytoca NCTC 13727
8118330620 16S ribosomal RNA Klebsiella oxytoca DSM 5175
8117964651 16S ribosomal RNA Klebsiella oxytoca NCTC 13727
8117964697 16S ribosomal RNA Klebsiella oxytoca NCTC 13727
8078247382 16S ribosomal RNA Klebsiella pneumoniae FF1010
8078245855 16S ribosomal RNA Klebsiella pneumoniae FF1010
2971714056 16S ribosomal RNA Klebsiella quasipneumoniae N18-04116
2969029708 16S ribosomal RNA Klebsiella quasipneumoniae N18-04101
2969034352 16S ribosomal RNA Klebsiella quasipneumoniae N18-04101
2969033893 16S ribosomal RNA Klebsiella quasipneumoniae N18-04101
2969034263 16S ribosomal RNA Klebsiella quasipneumoniae N18-04101
2969033554 16S ribosomal RNA Klebsiella quasipneumoniae N18-04101
2969034459 16S ribosomal RNA Klebsiella quasipneumoniae N18-04101
2971713920 16S ribosomal RNA Klebsiella quasipneumoniae N18-04116
8111663157 16S ribosomal RNA Klebsiella quasipneumoniae similipneumoniae 07A044T
2980390386 16S ribosomal RNA Klebsiella quasipneumoniae ATCC 700603
2980364483 16S ribosomal RNA Klebsiella quasipneumoniae ATCC 700603
8072397768 16S ribosomal RNA Klebsiella quasipneumoniae CAV2013
2686935600 16S rRNA. Bacterial SSU Klebsiella quasipneumoniae ATCC 700603
2879999479 16S ribosomal RNA Klebsiella quasipneumoniae CAV1947
2907909275 16S ribosomal RNA Klebsiella quasipneumoniae CAV2018
2980365290 16S ribosomal RNA Klebsiella quasipneumoniae ATCC 700603
2686939712 16S rRNA. Bacterial SSU Klebsiella quasipneumoniae ATCC 700603
2980363438 16S ribosomal RNA Klebsiella quasipneumoniae ATCC 700603
2980391432 16S ribosomal RNA Klebsiella quasipneumoniae ATCC 700603
2971713180 16S ribosomal RNA Klebsiella quasipneumoniae N18-04116
2686936435 16S rRNA. Bacterial SSU Klebsiella quasipneumoniae ATCC 700603
2980394769 16S ribosomal RNA Klebsiella quasipneumoniae ATCC 700603
2980391792 16S ribosomal RNA Klebsiella quasipneumoniae ATCC 700603
2971709502 16S ribosomal RNA Klebsiella quasipneumoniae N18-04116
2980363078 16S ribosomal RNA Klebsiella quasipneumoniae ATCC 700603
2971713537 16S ribosomal RNA Klebsiella quasipneumoniae N18-04116
2686939356 16S rRNA. Bacterial SSU Klebsiella quasipneumoniae ATCC 700603
2688552649 16S rRNA. Bacterial SSU Klebsiella variicola HKUOPLA
2880038652 16S ribosomal RNA Klebsiella quasipneumoniae HKUOPA4
8007807835 16S ribosomal RNA Klebsiella quasipneumoniae HKUOPJ4
2880039451 16S ribosomal RNA Klebsiella quasipneumoniae HKUOPA4
8007842961 16S ribosomal RNA Klebsiella quasipneumoniae HKUOPL4
8007843668 16S ribosomal RNA Klebsiella quasipneumoniae HKUOPL4
8007842768 16S ribosomal RNA Klebsiella quasipneumoniae HKUOPL4
8007843006 16S ribosomal RNA Klebsiella quasipneumoniae HKUOPL4
2880037282 16S ribosomal RNA Klebsiella quasipneumoniae HKUOPA4
2971710273 16S ribosomal RNA Klebsiella quasipneumoniae N18-04116
2880038786 16S ribosomal RNA Klebsiella quasipneumoniae HKUOPA4
8007807173 16S ribosomal RNA Klebsiella quasipneumoniae HKUOPJ4
8007841506 16S ribosomal RNA Klebsiella quasipneumoniae HKUOPL4
8007810625 16S ribosomal RNA Klebsiella quasipneumoniae HKUOPJ4
8007807495 16S ribosomal RNA Klebsiella quasipneumoniae HKUOPJ4
2880038740 16S ribosomal RNA Klebsiella quasipneumoniae HKUOPA4
8007843328 16S ribosomal RNA Klebsiella quasipneumoniae HKUOPL4
8007842292 16S ribosomal RNA Klebsiella quasipneumoniae HKUOPL4
8007812080 16S ribosomal RNA Klebsiella quasipneumoniae HKUOPJ4
